# Bayesian prediction of microbial oxygen requirement

**DOI:** 10.12688/f1000research.2-184.v1

**Published:** 2013-09-13

**Authors:** Dan B. Jensen, David W. Ussery

**Affiliations:** 1 Center for Biological Sequence Analysis, Technical University of Denmark, Lyngby, Denmark; 2 Comparative Genomics Group, Oak Ridge National Laboratory, Oak Ridge, TN 37831, USA

**Keywords:** Comparative genomics, oxygen requirements, prediction, Bayesian inference

## Abstract

**Background:** Prediction of the optimal habitat conditions for a given bacterium, based on genome sequence alone would be of value for scientific as well as industrial purposes. One example of such a habitat adaptation is the requirement for oxygen. In spite of good genome data availability, there have been only a few prediction attempts of bacterial oxygen requirements, using genome sequences. Here, we describe a method for distinguishing aerobic, anaerobic and facultative anaerobic bacteria, based on genome sequence-derived input, using naive Bayesian inference. In contrast, other studies found in literature only demonstrate the ability to distinguish two classes at a time.

**Results: **The results shown in the present study are as good as or better than comparable methods previously described in the scientific literature, with an arguably simpler method, when results are directly compared. This method further compares the performance of a single-step naive Bayesian prediction of the three included classifications, compared to a simple Bayesian network with two steps. A two-step network, distinguishing first respiring from non-respiring organisms, followed by the distinction of aerobe and facultative anaerobe organisms within the respiring group, is found to perform best.

**Conclusions:** A simple naive Bayesian network based on the presence or absence of specific protein domains within a genome is an effective and easy way to predict bacterial habitat preferences, such as oxygen requirement.

## Background

Identification of microbial organisms with specific habitat adaptations is important for a range of purposes, such as specifying organisms as likely producers of industrially or scientifically relevant enzymes. An easy-to-make prediction of adaptations to specific habitats based on genome sequences, independent of time consuming laboratory tests, would therefore be of value to researchers for narrowing down a list of potential organisms of interest for their particular purpose. In addition, a list of genomic features that effectively predicts the environmental preference of a group of organisms would aid scientific researchers in gaining a mechanistic understanding of the requirements a given environment imposes on its microbial inhabitants.

To demonstrate a method for making such predictions, this study aims to predict bacterial oxygen requirements. This choice was made in part because prediction and description of genomic characteristics relevant for oxygen requirements are relatively absent in the literature, in spite of a many characterized genomes available. Furthermore, when prediction of oxygen requirement has been attempted in the literature, the authors generally invoke the false dichotomy of a bacterium being either an aerobe or anaerobe ^1, 2^. Similar unfairly dichotic approaches are often seen with respect to other habitat classifications, *e.g.* salinity and thermophilicity ^3– 5^. In contrast, this study aims to distinguish between three different classifications: aerobe, anaerobe and facultative anaerobe. These oxygen requirement classifications can be found at the NCBI list of sequenced genomes ( http://www.ncbi.nlm.nih.gov/genomes/lproks.cgi) and have simple and specific definitions. Obligate aerobes are organisms that require the presence of oxygen for respiration, while the presence of oxygen is detrimental to the growth of obligate anaerobes. Non-obligate, or aero tolerant, anaerobes may grow in the presence of oxygen, but are unable to use it in respiration. In this study, we did not distinguish between these two types of anaerobes. Facultative anaerobic bacteria can use oxygen for respiration, but will also grow in the absence of oxygen, although typically more slowly ^6^.

Specific living-conditions will naturally impose selective pressures on the optimal set of protein functions, and a sensible basis for prediction would thus be the genomic make-up with respect to an organism protein domain profile. This idea has been the basis of a number relatively successful attempts at predicting different types of habitat adaptations ^1^.

For the purpose of classification prediction, this study implements a naive Bayesian classifier. This is a relatively simple method, but it has in the past been shown to be effective prediction tool in a vast range of areas, including bacterial thermophilicity prediction ^7, 4^, genetic risk factors for disease ^8, 9^ and taxonomic classification of fungi ^10^.

## Methods

### Selection of genomes

The genomes included in this study were selected from the NCBI genome database based on the oxygen requirement classifications in the NCBI Iproks table ( http://www.ncbi.nlm.nih.gov/genomes/lproks.cgi). To avoid overestimation of the predictive performance, only one member of each genus was randomly selected to be included within each classification. Thus the overall dataset configuration was as show in Table 5.

### Model construction

The included genomes where translated to predicted proteomes using the Prodigal tool ^11^ with default settings. The predicted proteomes were searched for the presence of the protein domain Pfam-A ^12^. This search was performed using hmmscan3 with default settings, a tool which is part of the HMMR3 package ^13^. The presence or absence of all Pfam-A domains found in the sum of proteomes was stored in a presence/absence matrix (Additional file 6). Based on this matrix, Pfam-A domains overrepresented in any one specific class were identified. Similarly to a previous study ^7^, overrepresentation is here defined as the domain being present in at least 65% of the members of a given class, and that the frequency in that class is significantly (p < 0.05) different from the frequency in all other classes, given a two-tailed independent *t*-test. In this manner, a list of class-associated Pfam-A domains, along with their frequency of occurring in each of the three classifications, was created. This list contained the observed likelihood of a given Pfam-A domain being present, given the classification, and will be referred to as the ‘likelihood file’. The script used to construct the model can be found in Additional file 7. All scripts can additionally be found at https://github.com/danbjensen/Oxygen_requirement_prediction. The scripts are also permanently available at http://dx.doi.org/10.5281/zenodo.7099.

### Prediction

Predictions were based on the above described likelihood file. A flat prior was used, meaning that initially the probability of an arbitrary genome being any of the three classifications was considered to be 1/N _Classes_ ~ 0.333. If a given domain was found to be present in a given genome, the probability of that genome belonging to each of the included classifications was updated by a factor of the observed likelihood for the individual groups, p(family|class). If the family or domain was found not to be present, the probability was updated by a factor of 1-p(family|class). The posterior probability for a given genome belonging to the various classifications, *C*, given the observed presence or absence of a specific domain, *O _i_*, was then calculated using Bayes rule:

$$p(C\text{|}O_{1-n})\;=\;\frac{\left(\Pi_{i=1}^{i=n}p(O_{i}\text{|}C)\right)\;\cdot \;prior(C)\;+\;PC}{\Sigma_{i=1}^{i=n}\;p\;(0_{i})}$$

**Figure 1.**
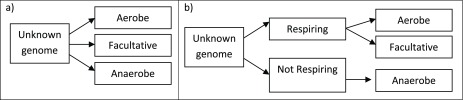
Schematic overview of the two methods used to predict oxygen requirement in bacteria. **a**) In the one-step prediction method the genomes in the test set are assigned a posterior probability for each of the three included classifications, given their protein domain profile. The genomes are predicted to belong to the classification to which they have the highest posterior probability. **b**) The genomes in the test set are first assigned posterior probabilities for being able or unable to respire, based on their protein domain profile. Using a second model, those genomes found most likely to be capable of respiration are assigned a posterior probability of belonging to the classifications Aerobe or Facultative.

A pseudo-count (PC) of 0.1 was used for all likelihoods to prevent the probabilities from plummeting to zero. The three included classifications of oxygen requirements were predicted using a one-step and two-step naive Bayesian inference network, as illustrated in Figure 1.

In the one-step approach, each genome is assigned the single classification it is considered most likely to have, based on its protein domain profile. The likelihood file is based on a training matrix containing the protein domain profiles of all genomes with their associated classification, with the exception of the genome being predicted (N-fold cross-validation).

In the two-step approach, every genome is first predicted to be able or unable to use oxygen for respiration. This is done based on a training matrix, containing every included genome, marked as either respiration-capable (aerobe and facultative anaerobe) or not respiration-capable (anaerobe). The genome being predicted is excluded from the training matrix. Any genome predicted to not be respiration-capable is considered to be a predicted anaerobe, while genomes predicted to be respiration-capable go through a second round of predictions. These predictions are based on the likelihood files derived from a matrix containing the protein domain profiles of aerobes and facultative bacteria only. Based on this, every genome predicted to be respiration-capable is predicted to be either an aerobe or facultative anaerobe. If a genome under prediction is present in the training matrix, the profile of this genome is excluded from the training. The script used to make the described predictions can be found as Additional file 8.

### Evaluation of predictive performance

To evaluate predictive performance, Matthew's Correlation Coefficient (MCC) ^14^ was used. As three categories were included in this study, the predictions for each category were evaluated individually by forcing the three classes into two; the one a given genome belongs to, and every other class. This is a common method for adapting the MCC method to prediction data with more than two possible classifications ^15^. The script used to calculate the performances can be found in Additional file 9.

## Results and discussion

### Predictive performance

To evaluate the efficiency of class-associated Pfam-A domains, *i.e.* Pfam-A domains found significantly more frequently in one specific oxygen requirement class compared to any other, as an input for a naive Bayesian classification of bacterial oxygen requirements, the Matthew's Correlation Coefficient (MCC) ^14^ was used. In the context of the MCC, a value of 1 indicates perfect correlation between predicted and actual class, a value of -1 indicates a perfect anti-correlation and a value of 0 is expected when the predictions are perfectly random. Two strategies were attempted: one where prediction of all three classifications was attempted in a single step and another where a simple Bayesian network was implemented, describing the oxygen requirement classifications as two nested dichotomies.

***One-step predictions***. Table 1 shows the predictive performance achieved when the three classifications are predicted in a single step, based on the relative abundance of the various Pfam-A domains in the different classes. The performance is clearly best for the prediction of aerobes and anaerobes, which perform with an MCC value well above 0, although not fully 1. The performance for predicting facultative anaerobes, although higher than 0, are not satisfyingly above what one might expect from random clustering of the data to be considered truly meaningful. The exact predictions and correct classification of the individual genomes are listed in Additional file 1.

**Table 1.** Predictive performance, measured in Matthew's Correlation Coefficient (MCC), achieved when using N-fold cross validation for one-step prediction. Predictions of all classes are performed better than random chance, although aerobe and anaerobe bacteria clearly show the best performance compared to facultative anaerobe bacteria.

Classification	Predictive performance (MCC)
Aerobe	0.63
Anaerobe	0.76
Facultative	0.31

To further examine the conditions behind the above performances, Table 2 shows the distribution of class-predictions for genomes of each of the three actual classifications. As can be seen, the vast majority of aerobe and anaerobe genomes are predicted correctly. For the facultative subsection of the dataset, however, many genomes are erroneously predicted to be aerobes. By contrast, the rate of erroneous prediction of genomes in these an aerobe or facultative anaerobe of being an anaerobe is rather low. This explains why the prediction performance of aerobe genomes appeared lower than for anaerobes; the false positive value in the MCC equation becomes larger for aerobe genomes, thus causing the overall value to drop.

**Table 2.** Overview of how the different classes are predicted, when using the one-step method. Aerobe bacteria are correctly predicted to aerobe in 87% of the cases and are mis-predicted to be facultative anaerobes in 11% of the cases. Similarly anaerobe bacteria are correctly predicted in 88% of the cases, and are mis-prediction of anaerobes as aerobe or facultative anaerobes happen equally frequently, in 6% of the cases. Facultative anaerobes are most commonly mis-predicted to be aerobes, in 44% of the cases. The facultative anaerobes are only correctly predicted in 35% of the cases.

	Aerobe genomes				Anaerobe genomes				Facultative genomes			
Predictions	Aerobe	137	87	%	Aerobe	6	6	%	Aerobe	43	44	%
	Anaerobe	3	2	%	Anaerobe	95	88	%	Anaerobe	21	21	%
	Facultative	17	11	%	Facultative	7	6	%	Facultative	34	35	%

This finding arguably makes sense in light of the fact that facultative anaerobes possess the ability to respire using oxygen, which is a feature missing in strict anaerobic organisms. It thus makes sense to assume that a specific list of enzymatic characteristics are required or useful for the organism to perform respiration, which one might thus expect to find in aerobes as well as facultative anaerobes. The same characteristics would likely not be useful in anaerobe bacteria, which would result in an enzymatic profile of aerobe and facultative anaerobe bacteria, which would stand out as separate from anaerobes.

***Two-step predictions***. Inspired by the findings described above, a reasonable prediction strategy would be to first separate the respiration-capable organisms from the anaerobes, and subsequently attempt to further distinguish between the two kinds of respiring bacteria. Here, the initial prediction of distinguishing anaerobe from non-anaerobe bacteria is based on the Pfam-A presence/absence data, with all aerobe and facultative anaerobe bacteria simply considered as the same classification. The secondary prediction is based on the Pfam-A presence/absence data from known respiration-capable genomes only, disregarding the anaerobe portion of the dataset. This two-step approach yields the overall predictive performances shown in Table 3. The exact predictions and correct classification of the individual genomes can be found in Additional file 2. It should be noted that a considerable improvement is found in the performance of prediction of facultative anaerobes. These improvements can be understood by how the members of the three classes are actually predicted, as shown in Table 4. Many facultative anaerobe genomes are still erroneously predicted to be aerobes. However, the percentage of correct predictions of aerobes and anaerobes has clearly increased compared to the one-step method, indicating that the two-step network offers some advantage.

**Table 3.** Predictive performance, measured in Matthew's Correlation Coefficient (MCC), of two-step Bayesian network for oxygen requirement prediction. The performance for aerobe and anaerobe predictions are the same as for the one step prediction method, but the performance for prediction of facultative anaerobes have increased from 0.31 to 0.39.

Classification	MCC
Aerobe	0.63
Anaerobe	0.76
Facultative	0.39

**Table 4.** Overview of how the different classes are predicted, when using the two-step method. Notice that the frequency of correctly predicted facultative anaerobes have not increased compared with the one-step method (33% vs. 35%), but that the fraction of erroneous predictions of aerobe and anaerobe bacteria have been decreased (5% vs. 11% for aerobes, 4% vs. 6% for anaerobes). Thus the better performance of the prediction of facultative anaerobe genomes is due to an increased accuracy in predicting aerobe and anaerobe bacteria rather than an increased accuracy in predicting facultative anaerobe bacteria.

	Aerobe genomes				Anaerobe genomes				Facultative genomes			
Predictions	Aerobe	141	90	%	Aerobe	8	7	%	Aerobe	47	48	%
	Anaerobe	8	5	%	Anaerobe	96	89	%	Anaerobe	18	19	%
	Facultative	8	5	%	Facultative	4	4	%	Facultative	32	33	%

**Table 5.** Number of genomes of the three different oxygen requirement classifications included in this study.

Classification	Number of included genomes
Aerobe	175
Anaerobe	112
Facultative	91

### Class-associated protein domains

As described above, the most effective method for predicting oxygen requirement attempted in the present study was the two-step Bayesian network, where anaerobes were first distinguished from the respiring bacteria (aerobes and facultative anaerobes). This study found a total of 252 protein domains to be consistently over-represented in anaerobe genomes, compared to the respiration-capable genomes. The specific likelihoods of these domains being present given an anaerobe or aerobe/facultative genome, are listed in Additional file 3.

Aerobe genomes were consistently distinguished from facultative anaerobe genomes by 402 domains, while facultative genomes consistently had 122 specific domains over-represented in their genomes, compared to aerobe genomes. The specific likelihoods of these 524 domains being present given that the genome is from an aerobe or facultative anaerobe bacterium, respectively, are listed in Additional file 4.

Thus, by applying the information provided in Additional file 3 and Additional file 4 in a two-step Bayesian estimation, as described in the Method section, it is possible to calculate the most likely oxygen requirement class of an arbitrary bacterial genome, provided a Pfam-A profile is available for said genome.

### Comparison to published prediction results

Very few studies that attempt to predict microbial oxygen requirements can be found in the literature. Two examples are the studies by Wu & Moore and Lingner *et al.* ^1, 2^. In both of these studies they distinguish just two classes of oxygen requirement at a time. Although Wu & Moore look at three classes (aerobe, anaerobe and facultative anaerobe), they only attempt dichotic predictions, always leaving out one class entirely. They are thus uninformative about the reliability of predictions where the genome in question can be any of the possible classifications. In contrast, the method described in the present study offer more realistic estimations of how well the prediction of any of the three included classifications will perform.

In their distinction of aerobic and anaerobic organisms, Wu & Moore report an average misclassification rate, when distinguishing between aerobe and anaerobe genomes, of 15% and 13% when basing predictions on Clusters of Orthologous Groups and KEGG Orthology groups, respectively. To allow for a direct comparison, the two-step method described in the present study shows an average misclassification rate of a slightly less than 8% (MCC = 0.84) when distinguishing between aerobe and anaerobe genomes alone (Additional file 5). This suggest that the two-step method described here, along with being more simple to perform, is actually almost twice as accurate, when directly compared to the methods presented by Wu & Moore.

Similar to the present study, Lingner *et al.* attempted to predict oxygen requirements based on protein domain profiles; however only the distinction between aerobe and anaerobe genomes was described. For this purpose, Lingner *et al.* reported a performance in the form of sensitivity multiplied by specificity, of 0.88, which is comparable to the 0.84 achieved for aerobe/anaerobe distinction when using the method described here (Additional file 5). To construct the protein domain profiles used by Lingner *et al.*, the number of each of the Pfam-A domains present in a given genome was used. In contrast, this study looked only on the presence or absence of the various Pfam-A domains in the individual genome. The comparable performances thus indicate that the presence of specific protein domains is indicative of oxygen requirements, regardless of the copy-number of those domains. Furthermore, it should be noted that Lingner *et al.* performed their predictions based on genomes available from NCBI 2009. They do not specifically specify the number of genomes labeled with respect to oxygen requirement at that time, but given the continuous additions of new genome sequences, it can reasonably be assumed to be fewer than the genomes available for the present study.

Data and scripts for Bayesian prediction of microbial oxygen requirement of selected bacteria from the NCBI genome database Additional file 1: Text-format (.txt). One-step prediction results. The classification predictions for all included genomes when using the one-step method and N-fold cross-validation.
Additional file 2: Text-format (.txt). Two-step prediction results. The classification predictions for all included genomes when using the two-step method and N-fold cross-validation.
Additional file 3: Text-format (.txt). Domains distinguishing anaerobes from respiring bacteria. The Pfam-A domains found significantly more frequently in bacteria which are capable of respiration (aerobes/facultative anaerobes) than in anaerobes, and vice versa.
Additional file 4 Text-format (.txt). Domains distinguishing aerobes and facultative anaerobes. The Pfam-A domains found significantly more frequently in anaerobe than facultative bacteria, and vice versa.
Additional file 5: Text-format (.txt). Predictions of aerobes and anaerobes only. The predictions of all of the included genomes which are either aerobe or anaerobe, excluding the facultative anaerobes. Made only to allow for direct comparison to results in the literature.
Additional file 6. Text-format (.txt). Protein domain presence/absence matrix. The presence/absence profiles with respect to Pfam-A domains of all included genomes.
Additional file 7: Python-format (.py). Get likelihoods from Pfam-domains. A python script used to identify the Pfam-A domains over-represented in one class compared to the others based on the training set, and on that basis construct the likelihood files used for predictions.
Additional file 8: Python-format (.py). Predictor. A python script used to predict the oxygen requirements classification of genomes in the test set, based on protein domain profile and the likelihood files created by Additional file 7.
Additional file 9: Python-format (.py). Predictive evaluations. A python script used to evaluate the predictors performance by calculating a Matthew's Correlation Coefficient for each of the classifications in the predictions made by Additional file 8. Click here for additional data file. Copyright: © 2013 Jensen DB and Ussery DW 2013 Data associated with the article are available under the terms of the Creative Commons Zero "No rights reserved" data waiver (CC0 1.0 Public domain dedication).

### Discussion of the Bayesian application used in this study

One of the main basic premises of the naive Bayesian inference method is that the various inputs, on which the inference is made, are mutually independent. In this study, the various inputs were different Pfam-A domains ^16^. Protein domains by definition consist of compact sequences that will fold, perform functions and even evolve independently of the rest of the protein in which they reside ^17^. Based on this fact, the basic premise of independence seems reasonable, and the method should thus be applicable.

Furthermore, even in situations where the premise of independence is invalid, the naive Bayesian classifier can be shown to produce excellent performance ^18, 19^. This means that even if certain protein domains might be found together regardless of classification, it would still be reasonable to expect the two-step method described here to be effectively applicable.

## Conclusions

The results presented in this study show that bacterial oxygen requirements can be accurately predicted without considering protein domain copy number. Although facultative anaerobes could be predicted with a performance significantly better than random guessing, further optimization is still desired so as to make the distinction meaningful in practice. Such optimization would include additional biologically meaningful markers, *e.g.* the presence of specific transcription factors. However, the distinction between aerobe and anaerobe organisms, are as good, or better than what is achieved by other methods published in the scientific literature ^1, 2^.

The best performances were achieved when using a simple Bayesian network, first distinguishing respiration-capable bacteria (aerobes and facultative anaerobes) from anaerobic bacteria, and subsequently distinguishing the aerobes from the facultative anaerobes. The respiration-capable bacteria could be distinguished from anaerobic bacteria with a Matthews' Correlation Coefficient of 0.76, while pure aerobes could be distinguished from anaerobes with a Matthews' Correlation Coefficient of 0.84.

Given the success with respect to distinguishing respiring bacteria from anaerobes, a reasonable follow up would be to study the class-associated Pfam-A domains identified in this study in more detail. They offer a logical first step for supplying a mechanistic model, explaining the genetic adaptations necessary for a bacterium given certain environmental oxygen exposures. Furthermore, we plan to test this method for prediction of other types of habitats, including cases with more than two categories. Based on the findings of this study, we would recommend that the categories of such cases be divided into biologically meaningful sets of dichotic super-classes, followed by two or more rounds of predictions.
