# Supplementary material for: Bayesian prediction of microbial oxygen requirement
Source: F1000Res. 2013 Sep 13;2:184. [Version 1] doi: 10.12688/f1000research.2-184.v1 (PMC4743139; doi:10.12688/f1000research.2-184.v1)
Supplement: Peer review history

# Peer review: Referee response for version 1

**DOI:** 10.5256/f1000research.2094.r3018

**Published:** 2014-01-30

**Authors:** Anita Krisko

**Affiliations:** 1 Mediterranean Institute for Life Sciences, Split, Croatia

The manuscript by Jensen and Ussery entitled *'Bayesian prediction of microbial oxygen requirement'* describes a novel improved method of distinguishing aerobic, anaerobic and facultative anaerobic bacteria. While previously published methods only demonstrate the ability to distinguish two classes at a time, the method described herein first distinguishes respiring from non-respiring bacteria, followed by distinguishing aerobic and facultative anaerobic bacteria within the respiring group.

There are several issues that require additional comments.

1. Looking at Additional Table 3 and Additional Table 4, I noticed that the definition of over-representation of domains may be a problem. For example, Additional file 3, line 6: domain ADK_lid is 86% represented in respiring bacteria and 94% in anaerobes. The authors decide that this domain is over-represented in anaerobes. However, isn't it over-represented in both? The authors state at the beginning of the manuscript that the over-represented domain is defined as the domain being present in at least 65% of the members of a given group. Have the authors considered defining over-represented domains as over-represented in one group and under-represented in the other? In any case, both have to be analyzed and commented on in the manuscript.
2. In addition to the abbreviation of each domain, it may be useful to include the function of each domain. Also, the authors need to discuss the functions of over-represented and under-represented (maybe even completely lacking) within each group of bacteria.
3. If the authors would like to claim this method for all organisms, the analysis needs to be performed also in archaea as well as the available eukaryotes. However, if the authors decide to present only the results on bacteria, this will have to be pointed out in the text.

I have read this submission. I believe that I have an appropriate level of expertise to confirm that it is of an acceptable scientific standard.

# Peer review: Referee response for version 1

**DOI:** 10.5256/f1000research.2094.r2198

**Published:** 2013-10-28

**Authors:** Kazuhiro Takemoto

**Affiliations:** 1 Department of Bioscience and Bioinformatics, Kyushu Institute of Technology, Fukuoka, Japan

The authors propose a prediction method for bacterial oxygen requirements using Bayesian instance with protein domain profiles. Although there is no originality in the context of prediction methods, the prediction accuracy of the proposed method is higher than that of the previous study. This suggests the validity of this method. This method is expected to be useful for estimating the oxygen requirement of newly isolated bacteria. The manuscript is generally well constructed, and it is readable. However, I have the following concerns:

- This study is limited to bacterial species. Thus, the authors have to emphasize this limitation, and the title should be “…bacterial oxygen requirements”.
- The authors have to discuss the prediction of archaeal oxygen requirement. For example, can this method be applicable to archaea?
- The authors should provide a clear description of the relationship protein domains and oxygen requirement. What protein properties are dominant for predicting oxygen requirement? A previous study (e.g. Kim KM *et al.*, 2012) may be helpful.
- I recommend that the authors mention some applications of the proposed method.
- The denominator in the first stand alone equation is incorrect: 0_i => O_i
- Are the numbers of included genomes in Table 5 correct? For example, Tables 3 and 4 include 157 aerobes, 108 anaerobes, and 98 (97 in Table 4) facultative anaerobes. This is inconsistent with Table 5.

I have read this submission. I believe that I have an appropriate level of expertise to confirm that it is of an acceptable scientific standard.
